# Artificial intelligence in the diagnosis of thyroid diseases: applications and challenges

**DOI:** 10.3389/fradi.2026.1740915

**Published:** 2026-04-01

**Authors:** Huayi Zhao, Tingyu Xue, Yan Zhang, Yanchao Lu, Danning Wang, Pei Yin, Lei Jiang, Dan Liu, Yong Wang

**Affiliations:** Department of Radiology and Nuclear Medicine, The First Hospital of Hebei Medical University, Shijiazhuang, China

**Keywords:** AI, classification, deep learning, detection, segmentation, thyroid, thyroid imaging modalities

## Abstract

Thyroid diseases, a prevalent class of endocrine system disorders, require diagnostic accuracy, which is essential for effective patient treatment and management. In recent years, artificial intelligence (AI) technology has made significant advancements in the medical field, providing new opportunities for the early diagnosis and precise treatment of thyroid diseases. This review discusses the latest applications of AI in the diagnosis of thyroid diseases, with a particular focus on the use of machine learning and deep learning (DL) algorithms in image classification, segmentation, and object detection within thyroid ultrasound, computed tomography, magnetic resonance imaging, and single photon emission computed tomography. Through the integration of cross-modal studies, this article reveals the application of AI across various imaging modalities, highlighting its potential value in feature extraction and risk stratification. Furthermore, we conduct an in-depth analysis of key challenges faced by AI applications, such as data heterogeneity (the decline in model performance due to data differences across institutions and equipment) and insufficient interpretability (DL models often function as “black boxes,” making it difficult to provide transparent decision-making rationale, which limits clinical trust and adoption). In summary, AI technology demonstrates notable advantages and developmental potential in the automated diagnosis of thyroid diseases; however, its clinical translation still requires addressing the aforementioned challenges. The resultant analysis demonstrates that AI holds promise in improving the diagnosis and treatment of thyroid diseases, offering new pathways for personalized medicine and better patient outcomes. Specifically, AI-driven tools can reduce diagnostic variability and errors in thyroid nodule assessment, enhance treatment precision with risk-stratified recommendations, and support more consistent, individualized clinical decisions.

## 1 Introduction

The global burden of thyroid disorders has risen markedly, with dysfunction recognized as the second most prevalent endocrine pathology worldwide. Epidemiological analyses reveal thyroid nodule detection rates of 19%–68% in adult populations (1), of which 5%–15% demonstrate malignant potential (2). Notably, since 1990, industrialized nations have experienced a marked upward trajectory in age-standardized incidence rates of thyroid cancer, positioning it among malignancies with the most rapid epidemiological escalation (3). This trend, reflected in approximately 449,000 and 137,000 new cases diagnosed in females and males globally in 2020 respectively, is strongly associated with advancements in high-resolution imaging modalities (4), characterized by superior diagnostic performance metrics [ultrasound accuracy: 92%–97%; computed tomography (CT) specificity: 85%–91%] (5–7). This diagnostic paradigm shift necessitates refined imaging stratification protocols to optimize benign-malignant differentiation and mitigate the risks of overdiagnosis inherent to contemporary screening practices.

Thyroid evaluation integrates multimodal imaging techniques, including ultrasound, CT, magnetic resonance imaging (MRI), and thyroid scintigraphy (8, 9). As the first-line modality, thyroid ultrasound combines gray-scale imaging and color Doppler flow imaging to provide structural and vascular details. At the same time, fine-needle aspiration biopsy (FNAB) is the gold standard for cytopathological confirmation (10). Advanced ultrasound techniques, such as ultrasound elastography, which quantifies tissue stiffness, and contrast-enhanced ultrasound, which further maps perfusion dynamics, can improve diagnostic accuracy (11, 12). In contrast to functional assessments, cross-sectional imaging modalities (CT/MRI) excel in anatomical delineation, with thin-slice CT reconstructions (≤1 mm) enabling precise monitoring of metastatic spread to cervical vasculature or aerodigestive tracts. Complementarily, thyroid scintigraphy provides metabolic profiling, demonstrating over 90% specificity in differentiating hyperfunctioning nodules (13). While each imaging modality offers distinct diagnostic advantages, their collective potential remains underutilized due to the lack of integrated AI frameworks capable of synthesizing information across modalities. The integration of AI across these diverse imaging modalities has progressed at different paces, reflecting each modality's unique technical characteristics and diagnostic roles. Ultrasound, as the most widely used first-line modality, has seen AI research predominantly focused on nodule detection, segmentation, and malignancy risk stratification using convolutional neural networks (CNN)-based architectures. For cross-sectional modalities such as CT and MRI, AI applications have focused on automated volumetry, precise anatomical segmentation, and radiomic feature extraction. In functional imaging such as thyroid scintigraphy, AI models have been developed to automate the quantification of radiotracer uptake. This modality-specific evolution of AI underscores that a “one-size-fits-all” approach is insufficient.

There is growing recognition that multi-modal learning—the simultaneous exploitation of complementary data sources—can substantially enhance model robustness and generalizability compared to single-modality approaches (14). In thyroid imaging, this presents an opportunity to fuse real-time morphological details from ultrasound, precise anatomical mapping from CT, and metabolic characterization from scintigraphy within unified DL architectures. Recent advances in multi-view deep feature learning from other neurological domains offer methodological insights (15). For example, frameworks that integrate time-domain and frequency-domain features from EEG signals have demonstrated superior performance in epilepsy detection, suggesting analogous potential for fusing multi-modal thyroid images.

However, the path to effective multi-modal fusion is fraught with modality-specific hurdles. First, heterogeneous data representation poses fundamental obstacles—ultrasound images, CT volumes, and scintigraphy functional maps exist in different dimensional spaces with distinct physical meanings, necessitating sophisticated alignment strategies. Second, modality-specific quality variability compounds these difficulties: while CT benefits from standardized Hounsfield units, ultrasound suffers from substantial operator-dependent variability. This disparity calls for modality-adaptive preprocessing and quality assessment mechanisms, such as embedding-based image quality evaluation frameworks that have shown promise in general medical imaging applications (14). Third, the computational demands of real-time multi-modal analysis may ultimately be met by next-generation communication infrastructures, such as 6G-enabled edge computing (16), which could facilitate distributed intelligence at the point of care.

Despite these technological advances, persistent challenges hinder diagnostic precision. Quantitative imaging features (e.g., echogenicity, enhancement patterns) frequently overlap between benign and malignant entities, with gray-scale ultrasound exhibiting false-positive rates of 14%–27% in indeterminate nodules (17, 18). Moreover, substantial interobserver variability persisted across thyroid imaging reporting systems (interobserver *κ* = 0.34–0.44, intraobserver *κ* = 0.33–0.54), underscoring the persistent operator-dependent limitations compounded by ambiguously classified echogenic foci (19). These uncertainties propagate significant clinical risks: junior radiologists exhibit 22% higher false-negative rates in malignant nodule identification than experts, potentially delaying critical interventions or prompting unnecessary FNAB, with 80% of biopsied nodules proving benign on histopathological evaluation (20).Therefore, developing standardized and intelligent diagnostic frameworks is imperative to mitigate overdiagnosis and optimize risk stratification.

To address these limitations, AI has emerged as a transformative tool for standardizing medical image interpretation (21–23). As a subset of computer science, AI leverages ML and DL algorithms to extract latent patterns from complex datasets, with CNNs achieving human-level performance in specific diagnostic tasks (24). DL models are primarily employed in thyroid image analysis for their superior feature extraction and pattern recognition capabilities (25). CNNs are characterized by hierarchical convolutional and pooling operations, progressively distilling features from low-level textures to high-level semantic information—such as nodule morphology, microcalcifications, and vascular patterns—forming the backbone of nodule detection and classification systems. For segmentation tasks, encoder-decoder architectures like U-Net, enhanced with skip connections, effectively integrate spatial details and semantic context to delineate gland and nodule boundaries (26). More recently, Vision Transformers (ViTs) have expanded the analytical scope by modelling long-range dependencies via self-attention mechanisms, enabling holistic assessment of nodules within their anatomical context (27).

**Figure 1:**
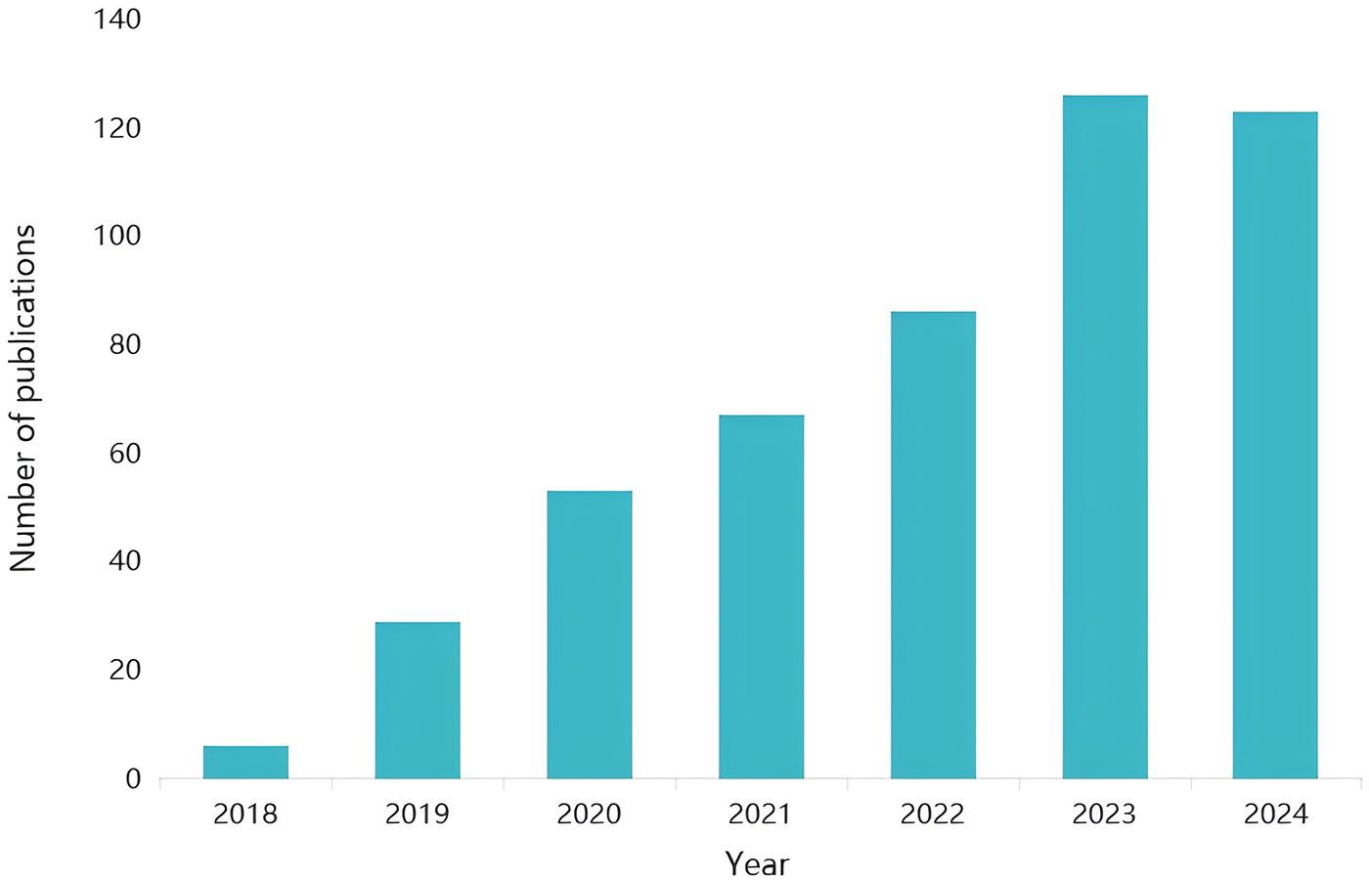
Number of articles indexed in pubMed® by year with title/abstract containing deep learning, thyroid, and imaging as keywords.

The clinical adoption of AI is driven by two synergistic factors: (1) the inherent complexity and diversity of medical imaging data (28), and (2) mounting evidence of AI's capability to mitigate diagnostic inconsistencies, for instance, reducing interobserver variability in thyroid nodule classification from *κ* = 0.42 to *κ* = 0.78 (29). In thyroid diagnostics specifically, algorithms (e.g., Inception-ResNet) trained on multi-institutional datasets achieve an Area Under the Curve (AUC) of 0.97 in nodule classification, outperforming human readers in reproducibility (30), while models like ThyGPT can reduce biopsy rates by over 40% while maintaining diagnostic sensitivity (31). Techniques such as transfer learning, attention mechanisms, and multimodal fusion are commonly employed to enhance model generalization across multi-institutional and multimodal data. Building on these advances, Haider et al. introduced Dual-OptNet—a hybrid deep learning architecture that effectively merges ResNet's skip connections with InceptionV3's multi-scale feature extraction—achieving 97% classification accuracy in thyroid disease diagnosis and establishing a foundation for subsequent multi-modal integration (32). These approaches mirror AI successes in other specialities; for example, DL models like ResNet-50 automate tumour grading in histopathology with over 90% accuracy (33, 34), CNN-based systems achieve high diagnostic performance in dermatology (35), and Food and Drug Administration (FDA) -approved AI tools screen diabetic retinopathy with sensitivity exceeding 99% (36). Nevertheless, challenges remain in thyroid AI applications, including sample dependence, limited interpretability, and performance inconsistency across heterogeneous datasets. Overcoming these limitations is essential to fully realize AI's potential in decentralizing expertise and reducing global healthcare disparities. The exponential growth of this field is underscored by the surge in annual publications on AI in thyroid disease, from six in 2018 to one hundred and twenty-two in 2024 (Figure 1).

This review aims to provide a detailed overview of emerging AI applications in the thyroid field, offering clinicians and researchers the latest insights into ongoing developments. This paper also summarizes the AI applications in multimodal imaging diagnostics for thyroid disorders, evaluating their research progress and clinical feasibility while delineating unresolved core challenges, including bias and generalizability in training data, standardized validation frameworks for model performance, and workflow integration barriers in clinical implementation. Looking ahead, the integration of quality-centric image assessment (14), multi-view feature learning strategies (15), and edge computing infrastructures (16) will be critical to realizing the full potential of multi-modal AI in thyroid disease diagnosis. Finally, this paper highlights AI's potential applications and possible directions in diagnosing thyroid diseases.

## 2 Basic theoretical knowledge of AI

AI, defined as “the engineering of intelligent systems capable of perceiving, reasoning, and acting autonomously” (37), aims to emulate human cognitive functions through computational algorithms (38). Its recent renaissance in medical applications is built upon three synergistic pillars: (1) the advent of Graphics Processing Unit-accelerated parallel computing systems, (2) the explosive growth of medical imaging data, and (3) paradigm-shifting innovations in deep neural network architectures (39).

**Figure 2:**
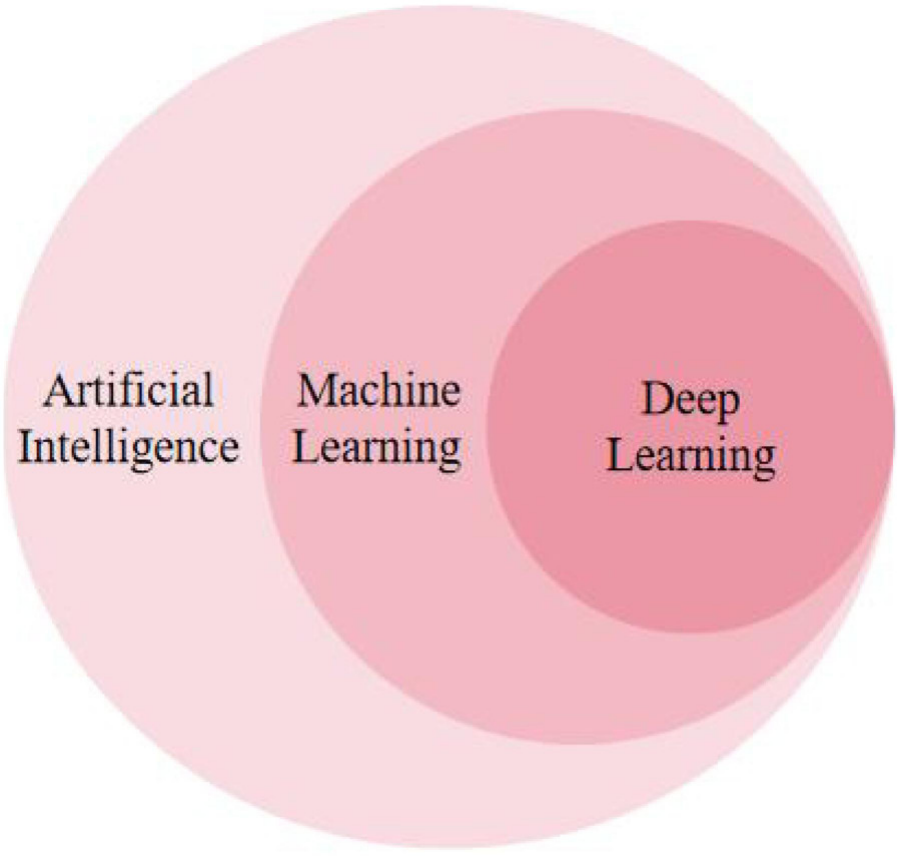
Hierarchical relationship between AI, machine learning, and deep learning. The diagram shows DL as a subset of ML, which is a subset of AI, providing the conceptual foundation for this review.

As a hierarchical field, AI encompasses various approaches to simulating intelligence. ML, a subset of AI, enables systems to learn patterns from data without explicit programming. DL, in turn, is a powerful branch of ML that utilizes multi-layered DNNs to process complex data and extract high-level features autonomously. The conceptual relationship among AI, ML, and DL is illustrated in Figure 2.

In medical image analysis, this hierarchy translates into distinct methodological approaches: ML typically relies on statistical models to decipher relationships between handcrafted imaging features and clinical outcomes, while DL employs hierarchical architectures to learn discriminative features directly from raw image data, enabling an end-to-end mapping from pixels to diagnostic decisions. This synergy is driving a paradigm shift from manual feature engineering toward data-driven intelligent analysis. The clinical impact of this shift is already measurable; for instance, the ThyNet model-assisted strategy demonstrably reduced FNAB rates from 61.9% to 31.2% while simultaneously lowering missed malignancy rates from 18.9% to 17% (40).

### 2.1 Machine learning

ML, a cornerstone of AI, enables systems to autonomously extract knowledge from data through iterative model optimization (41, 42). ML frameworks utilize algorithmic architectures (e.g., decision trees, neural networks) and large-scale datasets (*n* ≥ 10,000 samples) to execute classification, regression, and anomaly detection tasks with minimal human intervention.

**Figure 3:**
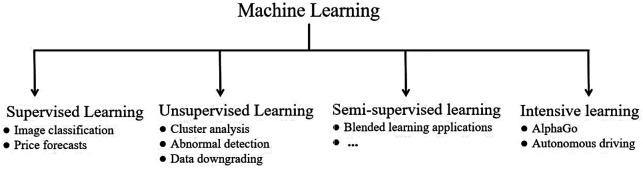
Four learning styles of machine learning.

Based on data annotation requirements, ML paradigms are categorized into four main types (Figure 3) (43–46).

#### 2.1.1 Supervised learning

Functioning as a digital “apprenticeship,” this is the most mature and widely used paradigm. Models are trained on expert-annotated datasets—such as images with delineated nodule boundaries or benign–malignant labels—to establish direct mappings between inputs and outputs. It has demonstrated excellent performance in thyroid disease tasks. For instance, by analyzing feature–label correlations from 29,000 annotated ultrasound images, the ThyNet-S model achieved senior radiologist-level performance in nodule classification (AUC = 0.93 vs. 0.91 for experts, *p* < 0.001) (47). Nevertheless, its scalability remains limited by the costly and time-consuming nature of large-scale annotations and the risk of inter-observer variability.

#### 2.1.2 Semi-supervised

This approach mitigates the annotation bottleneck by combining limited labeled data with abundant unlabeled data. The core principle is to extract rules from annotated samples and then infer intrinsic structures (e.g., clustering distributions) from unlabeled data, thereby enhancing model generalization. In thyroid ultrasound and CT analysis, this strategy reduces annotation burdens and improves adaptability across diverse devices and clinical settings. A representative graph neural network framework with attention mechanisms used pseudo-labeling to extract features from whole-slide images, enabling prognostic predictions for 3,950 patients across four cancer types and identifying biomarkers for risk stratification (48).

#### 2.1.3 Unsupervised learning

Requiring no prior labels, this paradigm identifies latent patterns or clusters within the data. In thyroid research, it has been applied to delineate molecular subtypes of thyroid carcinoma using clustering algorithms (e.g., k-means) and to detect outliers in genomic datasets (49), thereby uncovering novel biological insights that complement supervised approaches.

#### 2.1.4 Reinforcement learning (RL)

Diverging fundamentally from other paradigms, RL emulates a “trial-and-error” process where an agent learns to make decisions by interacting with an environment to maximize cumulative rewards. In thyroid imaging, RL holds promise for optimizing imaging protocols (e.g., autonomous ultrasound probe navigation) and enabling dynamic decision-making in real-time video analysis (50). Beyond imaging, RL has been explored in robotic thyroidectomy, to optimize surgical strategies for minimizing tissue damage and reducing operative time.

The development of performant ML models is inherently dependent on large-scale datasets and advancements in computational storage (51). Model efficacy further relies on meticulous algorithm selection, hyperparameter tuning, and feature engineering (52). As supervised learning remains the most prevalent paradigm in medical image analysis, it will be the primary focus of subsequent discussions in this review.

### 2.2 Deep learning

DL represents an advanced subset of ML, characterized by its ability to perform automatic feature extraction through deep, hierarchical neural networks—most notably Deep Convolutional Neural Networks (DCNNs) (53).

Inspired by biological neural systems, DCNNs employ multiple processing layers to iteratively learn and refine feature representations from raw input data. During training, connection weights between artificial neurons are optimized via backpropagation algorithmsfrom (54). This architecture enables a progressive feature abstraction hierarchy: initial layers capture low-level patterns (e.g., edges, textures in ultrasound), while deeper layers integrate these into high-level, semantically rich representations (e.g., specular microcalcifications or extracapsular invasion in thyroid nodules).

Compared to traditional ML, DL offers two key advantages: (1) Integrated Feature Learning: DL unifies feature extraction and classifier training within a single end-to-end framework, allowing the model to learn optimal features directly from the data itself, which often leads to superior performance. (2) Enhanced Model Capacity: DL models typically possess larger capacities and deeper architectures, enabling them to model complex, non-linear relationships in data and exhibit improved generalization to new, unseen examples.

**Figure 4:**
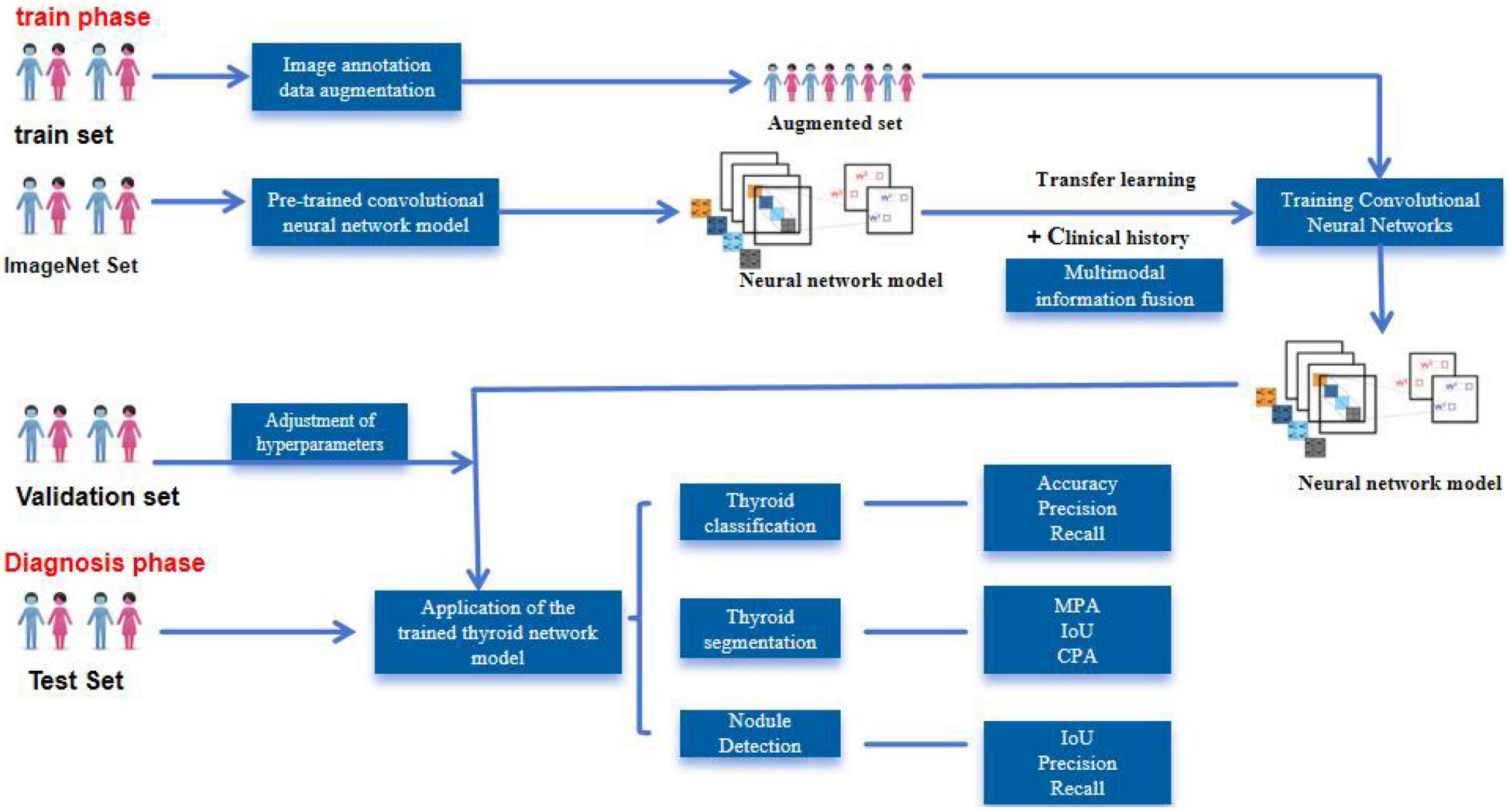
A workflow diagram for training, validation, and testing of deep learning model applications. MPA, mean pixel accuracy; IoU, intersection over union; CPA, class pixel accuray.

DL has revolutionized fields like computer vision and natural language processing (55). Its application in medicine, particularly in image analysis, has significantly advanced the performance of computer-aided diagnostic systems over classical ML methods. Figure 4 illustrates the standard pipeline for developing DL models, encompassing training, validation, and testing phases for tasks like classification, segmentation, and detection. In thyroidology, DL algorithms can automatically learn discriminative features from image data to enable automated diagnosis and classification. They can precisely locate and detect lesions (e.g., nodules) while performing detailed analysis of imaging characteristics (56, 57). The application of DCNNs has thus markedly enhanced the accuracy and efficiency of disease detection, representing a substantial advancement for clinical practice.

## 3 The application of AI in thyroid ultrasound

Ultrasound imaging is widely used for examining thyroid diseases due to its low cost, effectiveness, and lack of radiation. It is recommended by clinicians for the early detection and diagnosis of thyroid lesions (58, 59). However, the interpretation and description of ultrasound images often rely on the subjective judgment of ultrasound practitioners, requiring a high level of expertise and experience. Ultrasound practitioners typically need to interpret the features and changes in ultrasound images based on their clinical knowledge and professional judgment to determine the benignity or malignancy, size, shape, and location of thyroid lesions. Table 1 presents representative studies employing deep learning for Ultrasound imaging.

**Table 1:** Summary of deep learning studies in thyroid ultrasound imaging.

Author (year)	Dataset	Research content	Model	Performance
Li, et al. (2018). (126)	332,785 images	Diagnosis of thyroid cancer	ResNet50/Darknet19	Acc 0.889 Sens 0.922 Spec 0.856
Ko, et al. (2018). (127)	589 nodules	Nodule classification	DCNN	AUC 0.850
Buda, et al. (2019). (128)	1,377 nodules	Nodule classification	Faster R-CNN CNN	AUC 0.87
Zhu, et al. (2020). (74)	719 images	Nodule classification	TNet	Acc 0.865 AUC 0.861
Wang, et al. (2020). (73)	1,046 images	Diagnosis of thyroid cancer	Inception-Resnet-V2	Acc 0.873 Sens 0.842 AUC 0.901
Abdolali, et al. (2020). (60)	4,102 frames	Nodule detection	ResNet + Mask R-CNN	Prec 0.84 Sens 0.79 mAP 0.82
Yu, et al. (2020). (61)	2,000 images	Nodule detection	ResNet + Faster-RCNN	Acc 0.946 Sens 0.937 Spec 0.960
Bai, et al. (2020). (68)	13,984 images	Thyroid nodule risk stratification (TR1-TR5)	RS-Net	Acc 0.65
Koh, et al. (2020). (129)	15,375 images	Nodule classification	ResNet50/InceptionResNetV2	Acc 0.850 Sens 0.837 Spec 0.912
Han, et al. (2022). (130)	4,792 images	Nodule classification	DenseNet121	Sens 0.988 Spec 0.912
Wu, et al. (2022). (75)	1,736 images	Predicting thyroid lymph node metastasis	MMC-net	Acc 0.900 Sens 0.920 Spec 0.880
Yang, et al. (2022). (131)	508 images	Nodule classification	ResNet18	Acc 0.984 Sens 0.978 AUC 0.997
Görek, et al. (2023). (132)	1,254 images	Nodule classification	Multilayer RNN	Acc 0.999 Sens 0.999 Spec 0.999
Weng, et al. (2023). (133)	378 nodules	Nodule classification	ResNet101+ Faster R-CNN	AUC 0.69
Chen, et al. (2023). (30)	11,201 images	Nodule classification	Inception-ResNet + random forest	AUC 0.97 Acc 0.92 Sens 0.93
Sanaz, et al. (2024). (134)	983 patients	Nodule detection	Yolov5 + XGBoot	mAP 0.797
Yao, et al. (2024). (135)	88,796 images	Differentiate the metastatic cervical lymph nodes	ACE-Net	AUC 0.826
Zhao, et al. (2024). (136)	3,059 patients	Differentiate the metastatic cervical lymph nodes	Y-Net	Acc 0.825 Sens 0.848 Dice 0.832
Zhang, et al. (2024). (137)	518 patients	Tumor segmentation and lymph node metastasis prediction	FADLM	DSC 0.88 AUC 0.83
Zhang, et al. (2024) (138)	706 patients	Differentiate the metastatic cervical lymph nodes	MMD-DL	AUC 0.85 Acc 0.80 Spec 0.95
Wu, et al. (2025). (47)	35,008 images	Nodule classification	ThyNet-S	Acc 0.936 Sens 0.924 Spec 0.940
Xiang,et al (2025). (139)	2,764 images	Nodule segmentation	MAUNet	Dice 0.887–0.912
Zhang, et al. (2025). (140)	199 patients	Nodule classification	CNN	AUC 0.853 Acc 0.85
Chen,et al (2025). (141)	5,709 patients	Nodule classification	HTN-AI	Dice 0.91 Acc 0.92 Spec 0.99
Agyekum,et al (2025). (142)	264 images	Predicting the efficacy of ultrasound-guided microwave ablation for benign thyroid nodules	EfficientNetb1-fine tune	AUC 0.85 Acc 0.79

This table summarizes 25 studies published between 2018 and 2025 that apply deep learning algorithms to thyroid ultrasound analysis. For each study, we list authors and year, dataset characteristics, research task (e.g., nodule detection, segmentation, malignancy classification), model architecture, and key performance metrics. The table is organized chronologically to illustrate the evolution of the field. Acc, accuracy; Sens, sensitivity; Spec, specificity; AUC, area under the ROC curve; Dice, dice coefficient; DSC, dice similarity coefficient; mAP, mean average precision.

### 3.1 Automatic detection of thyroid nodules

**Figure 5:**
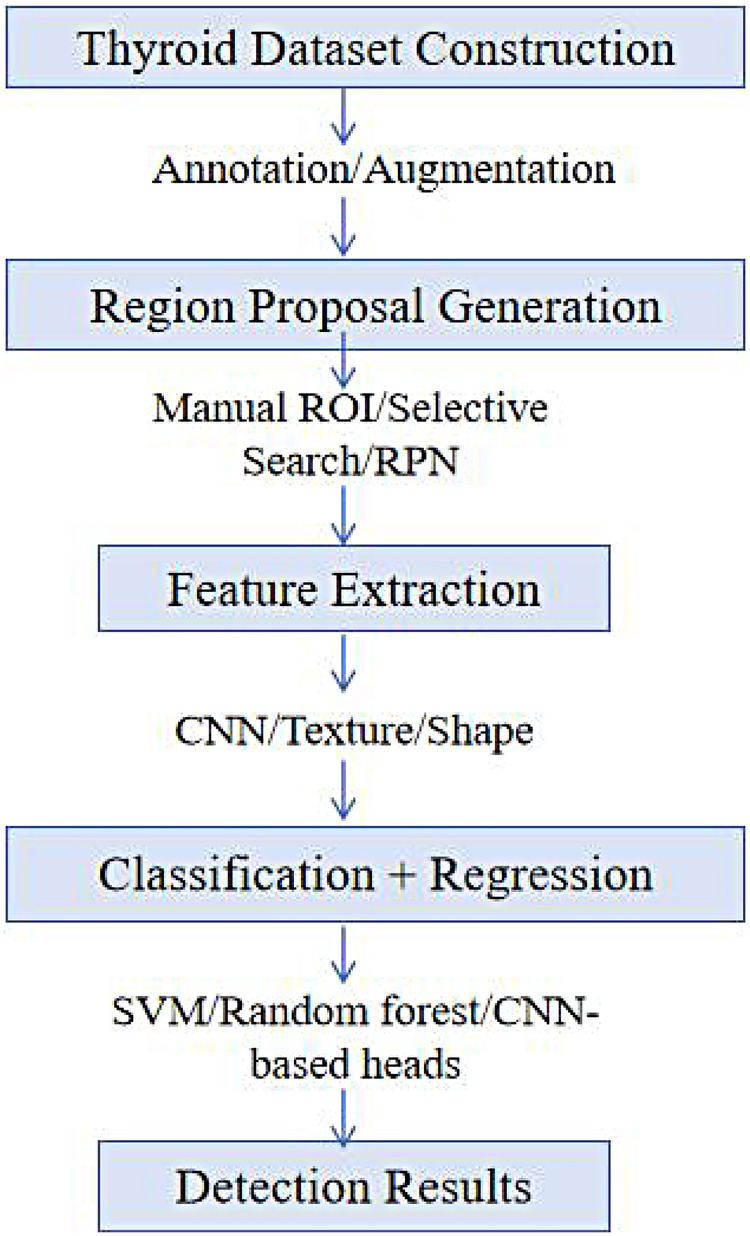
Detection workflow for thyroid nodules. ROI, region of interest; RPN, region proposal network; CNN, convolutional neural network; SVM, support vector machine.

With the continuous optimization of DL algorithms, thyroid nodule detection technology has achieved significant progress in recent years. The workflow for thyroid nodule detection typically consists of four stages: dataset construction, candidate region generation, feature extraction and selection, and candidate region classification (Figure 5). Much of the detection work is based on two-dimensional thyroid ultrasound datasets. For instance, Abdolali et al. (60) developed a DL framework for thyroid nodule detection using a multitask model, Mask R-CNN. The framework achieved detection accuracy, recall, and mean Average Precision (mAP) values of 84%, 79%, and 82%, respectively, outperforming the existing state-of-the-art detection methods. Yu et al. (61) constructed a Faster-RCNN model with a ResNet backbone, achieving remarkable performance in terms of accuracy, sensitivity, and specificity (94.6%, 93.7%, and 96.0%, respectively), significantly surpassing traditional machine learning algorithms like support vector machines (SVM) and standalone Faster-RCNN models. Chen et al. (62) employed the Yolov5 model to train and test on over 30,000 thyroid-analysis ultrasound images. The model achieved 77.81% and 73.33% accuracy in distinguishing echogenic lesions (calcification or colloid) across two datasets, far exceeding radiologists’ 61.29% and 59.38% accuracy. Qi et al. (63) used the Mask-RCNN18 network model to train on ultrasound images, integrating both semantic segmentation and object detection tasks. The model achieved an AUC of 0.91 in the internal validation set and 0.88 in the external validation set. Ma et al. (64) proposed a DL framework (YOLOv3-DMRF) for thyroid nodule detection and recognition, achieving mAP values of 90.05% and 95.23% on two test sets.

### 3.2 Classification of benign and malignant thyroid nodules

Thyroid nodules are the most common thyroid disease (65), with a study on healthy populations reporting a detection rate as high as 47% through ultrasound examination (66). The widespread use of various imaging modalities has contributed to the increased detection of thyroid nodules. Although most thyroid nodules are benign, physicians will further consider evaluation and screening to rule out the risk of malignancy for patients with detected nodules. In thyroid ultrasound examinations, most computer-aided diagnosis (CAD) systems aim to differentiate between benign and malignant thyroid nodules. The classification features commonly used in AI models are derived from the American College of Radiology's Thyroid Imaging Reporting and Data System, including size, shape, echogenicity, margins, and calcification (67). These are the same features that clinicians rely on for final diagnosis.

Over the past decade, various AI models have been developed to achieve risk stratification, refinement, and automated interpretation of thyroid nodules using ultrasound images or their derived features, thereby reducing unnecessary fine-needle aspiration rates (68, 69). For example, Zou et al. developed an age-stratified DL model, ASMCNet, for classifying thyroid nodules and trained and validated it using retrospective data comprising 10,391 ultrasound images from three hospitals. On the test set, the model achieved an AUC of 0.906, a sensitivity of 86.1%, and a specificity of 85.1%, significantly outperforming traditional non-age-stratified models and radiologists at various experience levels (70). Overall, DL algorithms have demonstrated significant improvements in specificity and accuracy, mainly due to their capacity to capture complex patterns in the data.

Given the significant heterogeneity in model design and training datasets, comparing the actual performance of models and assessing their accuracy in real-world settings remains a highly challenging task. Many well-established models with external validation have demonstrated experimental results on different datasets. Several studies have highlighted that the use of AI algorithms can significantly enhance the diagnostic performance of junior radiologists, improving their accuracy to a level comparable to that of senior radiologists (71, 72).

Moreover, several studies have found that when AI models are used as auxiliary diagnostic tools, they can enhance radiologists’ performance, with AUC values ranging from 0.86 to 0.90 (73, 74). It is essential to note that using different datasets, model designs, and performance evaluation methods in these studies may impact the outcomes. Additionally, these findings do not fully represent the performance of AI models in all scenarios or real clinical settings. Therefore, further research and validation are essential to assess AI's accuracy, reliability, and clinical applicability in real-world environments.

### 3.3 Other applications

In addition to thyroid nodule classification and object detection, several studies have also focused on predicting thyroid lymph node metastasis, risk stratification of thyroid nodules, and differential diagnosis of thyroid diseases. For instance, Wu et al. (75) designed a multimodal neural network (MMC-net), achieving an 86.0% to 90.0% accuracy across two independent validation sets, significantly outperforming three single-modality DL networks. Chang et al. (76) developed an AI-based DL model to predict cervical lymph node metastasis in thyroid cancer, achieving AUCs of 0.809 and 0.829 in internal and external validation groups, respectively. Bai et al. (68) proposed a DL-based method for automatic thyroid nodule risk stratification, named the Risk Stratification Network (RS-Net), which achieved a risk stratification accuracy of 65% and a benign-malignant classification accuracy of 88%. These studies aim to provide more detailed and comprehensive assessments of thyroid diseases, offering physicians additional information for treatment decision-making. It should be noted, however, that these studies are still in their early stages, and further validation and evaluation are necessary to assess the effectiveness and clinical applicability of DL models. These studies present new perspectives and potential opportunities for treating and prognosing thyroid diseases.

## 4 The application of AI in thyroid CT

### 4.1 Classification of benign and malignant thyroid nodules

Although ultrasound is the preferred method for diagnosing thyroid diseases, CT plays a vital role in determining the nature of thyroid nodules and in the pre- and post-assessment and treatment of malignant tumors. Thyroid nodules typically appear as low-density areas on CT images. Benign nodules, such as adenomas, generally present with a well-defined capsule and clear margins, whereas malignant nodules exhibit infiltrative growth with indistinct capsules. Consequently, both benign and malignant nodules can result in alterations to the internal structure and tissue density of the thyroid on CT images, serving as a basis for diagnosing thyroid nodules. CT examinations are less dependent on operator experience and have greater reproducibility, but due to resolution limitations, they are only suitable for nodules with a maximum diameter greater than 5 mm.

Additionally, the information richness of CT images is relatively high, making simple visual analysis insufficient to capture deeper characteristics of lesions, thus falling short of the demands of precision medicine and personalized treatment. AI methods can quantify the relationships between pixels and spatial distributions in CT images, fully exploiting hidden information that is not visible to the naked eye. This information can be presented quantitatively and objectively, guiding clinical diagnosis, treatment, and prognosis. AI technology has ushered CT image analysis into a new phase. By extracting subtle features from images, AI can assist physicians in detecting potential lesions earlier and providing a basis for developing personalized treatment plans. Moreover, AI can integrate other clinical data for more complex and comprehensive analyses, enhancing the accuracy and reliability of diagnoses. Deep learning approaches applied to CT are detailed in Table 2.

**Table 2:** Summary of DL studies in thyroid CT imaging.

Author (year)	Dataset	Research content	Model	Performance
Lee, et al. (2019). (83)	3,838 images	Lymph node classification	Xception	Acc 0.828 Sens 0.802 Spec 0.830
Lee, et al. (2019). (84)	995 images	Lymph node classification	ResNet50	Acc 0.904 Sens 0.904 Spec 0.904
Zhao, et al. (2021). (77)	986 nodules	Nodule classification	Ensemble mode	AUC 0.947 Acc 0.859 Sens 0.919
Jin, et al. (2022). (143)	293 lymph nodes	Predicting lymph node metastasis	Combined model	AUC 0.890 Sens 0.915 Spec 0.757
Zheng, et al. (2023). (144)	113 lymph nodes	Predicting lymph node metastasis	ResNet	AUC 0.824 Sens 0.839 Spec 0.769
Wang, et al. (2023). (85)	671 patients	Predicting lymph node metastasis	AI system	AUC 0.84
Wang, et al. (2023). (145)	574 images	Lymph node detection and classification	A-ResNet50-W	Acc 0.96 AUC 0.894
Li, et al. (2024). (146)	281 lymph nodes	Lymph node classification	VGG19	AUC 0.86 Acc 0.88 Sens 0.83
Yu, et al. (2024). (147)	1,266	Predicting lymph node metastasis	RefineNet	AUC 0.910 Sens 0.848 Spec 0.881

This table summarizes 9 studies published between 2019 and 2024 that apply DL algorithms to CT analysis. For each study, we list authors and year, dataset characteristics, research task (e.g., nodule detection, segmentation, malignancy classification), model architecture, and key performance metrics. Acc, accuracy; Sens, sensitivity; Spec, specificity; AUC, area under the ROC curve.

Deep learning models based on CT images can further improve the accuracy of diagnosing benign and malignant thyroid nodules. Zhao et al. (77) employed five commonly used CNN and ensemble models to differentiate between benign and malignant thyroid nodules in CT images. The results showed that the AUC for diagnosing malignant tumors using these five models and the ensemble model ranged from 0.901 to 0.947, significantly outperforming our radiologists (*P* < 0.05). Li et al. (78) designed an end-to-end automatic recognition and classification system for thyroid nodules based on CNN, consisting of an Eff-Unet segmentation network and a CNN-F classification network. The segmentation IOU reached 0.855 in the test set, and the classification accuracy was 85.92%. Zhao et al. (79) used an improved U-Net fully convolutional network architecture to segment regions of interest (ROI) in the thyroid and employed a fused convolutional neural network to detect malignant nodules from both ROI and original images, achieving an accuracy of 95.73%. Zhang et al. (80) proposed a multi-channel convolutional neural network structure, achieving recognition accuracies of 97.2% and 97.4% for left and right thyroid datasets from CT images, respectively. Li et al. (81) developed a Deep learning-based model to predict malignant lymph nodes in thyroid cancer patients, even when no suspicious features were visible on conventional CT images. The VGG16 model, applied to small field-of-view (FOV) CT images, demonstrated higher accuracy (86%) and sensitivity (88%) than models based on spectral parameters, with an AUC of 0.89. This suggests the VGG16 model offers a noninvasive and effective approach for predicting malignant lymph nodes without suspicious CT features.

### 4.2 Prediction of thyroid lymph node metastasis

Cervical lymph node metastasis (LNM) is a significant factor contributing to poor prognosis in patients with thyroid cancer (82). CT imaging can effectively assist in determining the presence of lymph node metastasis, enabling early intervention and effective treatment, which is crucial for improving the survival rates and quality of life of thyroid cancer patients. Therefore, early assessment of deep cervical lymph nodes and distant metastasis is essential for the precise treatment of thyroid cancer patients.

Deep learning-based CT prediction of thyroid lymph node metastasis represents an advanced technology developed from benign and malignant classification, offering significant value in diagnosing lymph node metastasis in thyroid cancer. Lee et al. (83) evaluated the performance of eight DL models using preoperative CT images from 3,838 thyroid cancer patients. The Xception model, which performed the best, achieved an accuracy of 82.8%, a sensitivity of 80.2%, and a specificity of 83.0%. This CAD system also demonstrated potential for improving diagnostic specificity and accuracy, especially in cases where previous models performed poorly. Building on prior research, Lee et al. (84) utilized 995 CT images for prediction, with the ResNet-50 model achieving an accuracy of 90.4%. The model highlighted key areas for further clinical investigation using attention heatmaps. Wang et al. (85) developed an AI system combining DenseNet with convolutional attention modules for predicting preoperative cervical lymph node metastasis (CLNM) in papillary thyroid cancer (PTC) based on CT images. Compared to traditional methods, the AI system excelled in predicting CLNM and significantly improved radiologists’ accuracy, potentially enhancing personalized clinical decision-making. Zheng et al. (86) developed a three-dimensional ensemble model based on ResNet for distinguishing between benign and metastatic lymph nodes in PTC using CT images. The model achieved an AUC of 0.824, with a sensitivity of 83.9% and specificity of 76.9%, and shows promise for clinical application in preoperative assessment.

### 4.3 Other applications

He et al. (87) developed a high-accuracy, high-efficiency DL method for delineating non-contrast head and neck CT images. This method demonstrated exceptional accuracy and robustness through retrospective Analysis of CT images from 1,977 suspected thyroid cancer patients. The testing results showed significant improvements in Dice similarity coefficient, Jaccard index, sensitivity, and specificity. Wu et al. (88) developed a deep multimodal learning network to integrate various clinical contexts and imaging modalities into the algorithm to enhance the prediction accuracy of lymph node metastasis in primary thyroid cancer patients. This method achieved an average F1 score of 0.888 and an AUC value of 0.973 across two independent validation sets, significantly outperforming single-modality DL networks. The results indicate that this multimodal network relies more on image data for predictions, providing radiologists with a powerful tool and a deeper understanding of the condition.

## 5 The application of AI in thyroid MRI

MRI offers significant advantages in the evaluation of thyroid diseases. It enables a comprehensive assessment of the extent of lesions and their relationship with surrounding critical structures through multiparametric and multidimensional imaging. MRI includes various imaging modalities that reflect different characteristics of lesions. In addition to high soft tissue resolution, diffusion-weighted imaging and apparent diffusion coefficient can reveal the microscopic structural characteristics of lesions, such as cellular density and membrane integrity, which are particularly advantageous for diagnosing malignant tumors. Recently, the application of AI has introduced new possibilities for further optimizing MRI. AI technology allows for the integrated Analysis of the rich information in different MRI imaging modalities, extracting deep features that are not readily visible to the human eye. This approach not only enhances the early diagnostic capabilities for thyroid diseases but also aids in more accurately assessing the nature and clinical significance of lesions. Therefore, combining the multiparametric imaging characteristics of MRI with AI technology offers more comprehensive and precise support for diagnosing and treating thyroid diseases, representing a significant direction for future development.

Wang et al. (89) utilized 120 cases of pathologically confirmed papillary PTC patients, employing the Least Absolute Shrinkage and Selection Operator for radiomic feature selection, and combined it with a gradient boosting classifier for classifying PTC invasiveness, achieving an AUC of 0.92. This study demonstrates that machine learning-based multiparametric MR imaging radiomics can accurately distinguish between invasive and noninvasive PTC preoperatively. Naglah and colleagues (90) proposed a novel CAD system based on multimodal MRI to differentiate between benign and malignant thyroid nodules. This system was compared with recent CNN models and several machine learning frameworks using handcrafted features. The CAD system achieved a diagnostic accuracy of 0.87, specificity of 0.97, and sensitivity of 0.69, outperforming all comparison methods in performance. Sharafeldeen et al. (91) A study involving 55 patients with thyroid nodules was conducted, utilizing an artificial neural network system to integrate functional imaging features (apparent diffusion coefficient) from T2-weighted MRI with morphological and texture features. The results indicated that the fusion system incorporating all biomarkers achieved high accuracy rates ranging from 92.9% to 95.5%, demonstrating significant potential in differentiating thyroid nodules and assessing their benign or malignant nature.

## 6 The application of AI in thyroid single-photon emission computed tomography (SPECT)

Compared with ultrasound, CT, and MRI images, SPECT offers certain unique advantages in thyroid diagnosis. ^99m^TcO4^−^ thyroid scintigraphy is the primary method used to assess the thyroid gland's location, shape, size, and functional status, providing crucial guidance for treating various thyroid disorders (92). With the continuous development of AI technologies, the application of AI to thyroid SPECT image analysis has significant potential. Through techniques such as DL, AI can enable automated thyroid SPECT image analysis, thereby improving diagnostic accuracy and efficiency. For instance, AI can assist clinicians in rapidly locating lesions, reducing diagnostic time, and minimizing the risk of missed diagnoses. Moreover, AI can facilitate personalized diagnosis and treatment recommendations for thyroid diseases, offering more precise medical services for patients.

Ma et al. (93) employed an optimized DenseNet CNN architecture for computer-aided diagnosis of three thyroid diseases: Graves’, Hashimoto's, and subacute thyroiditis. The experimental results demonstrated that this approach outperformed other neural network algorithms. Yang et al. (94) constructed an AI system using four pre-trained neural networks to automatically classify four patterns in thyroid scintigraphy images. The results showed that all four pre-trained neural network architectures achieved an overall classification accuracy of over 90% for the four common uptake patterns in thyroid scintigraphy, with the best-performing model, InceptionV3, achieving an overall accuracy of 92.73% on the internal validation set and 87.75% on the external validation set. Qiao et al. (95)reported that DL models (i.e., AlexNet, VGG-16, and ResNet) exhibited good diagnostic performance in thyroid scintigraphy, with the accuracy and specificity of all three models exceeding 85%. The experimental results suggest that these DL models could be effective tools for nuclear medicine residents in diagnosing Graves’ disease and subacute thyroiditis. Our previous experimental study (96) also yielded satisfactory results, with the DCNN model showing higher sensitivity and specificity in identifying Graves’ disease, subacute thyroiditis, and thyroid tumors compared to nuclear medicine physicians. The modified ResNet-34 model performed the best, achieving an accuracy of 94.4% and a sensitivity of 94.5% on the internal validation set, demonstrating the feasibility of using DL models to assist clinicians in improving diagnostic efficiency. Chen et al. (97) collected images from 70 SPECT thyroid patients, the proposed CNN algorithm achieved a higher Dice score than the backpropagation neural network algorithm, showing relatively better segmentation performance. It accurately localized the anatomical information of thyroid diseases and could potentially replace traditional diagnostic methods for thyroid disease diagnosis. The Kwon team (98) developed a deep learning-based non-CT quantitative SPECT for measuring thyroid uptake percentage. Experimental results showed that the AI-generated *μ*-maps were almost identical to the original real μ-maps, and the automated thyroid segmentation was highly consistent with manual thyroid segmentation, further confirming the reliability of AI. Guo et al. (99) designed a CAD system using a ResNet-18 pre-trained model to improve the classification accuracy of thyroid tissue remnants, aiming to verify the clinical utility of the CAD system in diagnosing thyroid tissue remnants. The fine-tuned model achieved an impressive accuracy of 96.69% and sensitivity of 94.75%, significantly outperforming other models. Kavitha et al. (100) developed a multilayer fully connected deep network using the RxWBSs dataset to automatically locate and identify metastatic lymph nodes (MLN) in thyroid residual tissues. This study used image patches and pixel location probability changes of MLN and residual tissues as inputs for the neural network. Through this efficient automatic method, the model achieved a high F1 score in detecting MLN, outperforming physician ratings (*P* < 0.001).

## 7 Discussion

### 7.1 Considerations for model selection and case analysis

In the automated Analysis of thyroid ultrasound images, different models exhibit substantial differences in task objectives, dependency on training data, and clinical applicability. Two-stage instance segmentation models, such as Mask R-CNN, adopt a “detection–segmentation” workflow in which nodules are first localized and then assigned pixel-level masks (101). This approach enables delineation of nodule boundaries and subtle structures, making it suitable for high-precision tasks such as volumetric monitoring and radiomics analysis. For example, Liu et al. (102) reported a Dice coefficient of up to 0.91 on an internal dataset. However, these models rely heavily on large-scale annotated data, have high computational complexity, and exhibit slow inference speed, posing challenges for real-time clinical applications and cross-center generalization.

In contrast, single-stage detection models, such as the YOLO family and SSD, achieve rapid localization through end-to-end regression (103). These architectures offer significantly faster inference times and leaner computational requirements, making them suitable for real-time screening applications and diagnostic support systems. Yang et al. (104) demonstrated that YOLOv5 achieved 90.1% detection precision while processing dozens of images per second. The trade-off for this efficiency comes in the form of bounding-box-only localization without pixel-accurate segmentation capabilities, often resulting in reduced sensitivity for small or low-contrast nodules.

Classification performance depends critically on both model architecture and input data characteristics. Whole-image input models capture comprehensive contextual information and background features, facilitating effective training even with limited samples. In contrast, models trained on precisely segmented nodule regions focus exclusively on intrinsic features such as echogenicity, calcification patterns, and margin characteristics, consistently demonstrating superior classification accuracy and specificity across multiple studies (105). Transformer-based architectures show particular promise for handling heterogeneous datasets due to their enhanced global feature modeling capabilities, though their application in thyroid imaging remains preliminary. Future developments should prioritize lightweight hybrid detection-segmentation networks and advance weakly supervised learning approaches to reduce annotation dependencies. Multimodal integration, combining imaging with clinical and laboratory data, will be essential for enhancing both generalizability and clinical relevance.

The systematic evaluation presented in Table 3 provides practical guidance for model selection and clinical implementation while identifying promising research directions. Specifically, the comparison reveals that ResNet-based architectures remain dominant in classification tasks due to their balance between representational capacity and training stability on small-to-moderate-sized medical datasets. For instance, a recent study comparing nine pre-trained CNNs on a public thyroid ultrasound dataset found that ResNet50 achieved the best performance, with an accuracy of 96.9% and an AUC of 0.97, outperforming other architectures, including EfficientNetB0 (accuracy 93.09%, AUC 0.94) (106). The authors attributed this success to ResNet's residual connections, which effectively mitigated gradient vanishing even with limited annotated images.

**Table 3:** Presents the performance differences among various models and their applicable scenarios.

Model	Task objective	Strengths	Limitations	Application scenarios
Mask R-CNN	Nodule segmentation and detection	High-precision pixel-level segmentation with excellent boundary delineation, suitable for fine structural Analysis.	High computational complexity, slow inference speed, and strong dependence on large-scale, high-quality annotations	Research applications, nodule morphology monitoring, and radiomics Analysis.
YOLO	Nodule detection	Fast inference speed and lightweight architecture make it suitable for real-time applications	Lacks pixel-level segmentation and morphological quantification; reduced sensitivity to small or low-contrast nodules.	Real-time screening, computer-aided diagnosis, and preliminary localization.
U-Net	Nodule segmentation	High segmentation accuracy with strong local detail restoration, suitable for small-sample learning.	Computationally demanding and still dependent on sufficiently annotated large-scale datasets.	Precise medical image segmentation and lesion localization.
ResNet	Nodule classification	Strong, deep feature extraction capacity, suitable for whole-image classification.	Performance strongly depends on accurate segmentation of regions of interest.	Benign–malignant nodule classification and global image analysis.
Transformer-based	Global feature modeling	Captures global contextual information and performs well on diverse and complex tasks.	High computational cost, long training times, and application stability remain to be validated.	Multimodal fusion, long-range dependency modeling, and large/complex datasets.
EfficientNet	Nodule classification and detection	Lightweight and efficient, with fewer parameters and fast inference, it balances accuracy with computational cost.	May underperform compared with more complex architectures in certain tasks.	Resource-constrained clinical settings and large-scale data applications.

In contrast, EfficientNet variants are often deployed in resource-constrained settings where inference speed is prioritized. MobileNetV3, for example, has been evaluated alongside ResNet50 in thyroid follicular neoplasm classification, demonstrating its viability for clinical applications where computational efficiency is paramount. However, performance trade-offs exist:while ResNet50 achieved strong results in multi-center follicular tumor differentiation, the choice between architectures must balance accuracy against deployment constraints.

For the segment, U-Net's encoder-decoder architecture remains preferred for precise boundary delineation. Recent advances have further enhanced this architecture: the HFA-UNet model, which combines hybrid and full attention mechanisms with a U-Net-like structure, achieved significant improvements on public thyroid datasets, with Dice score increases of 2.36% on DDTI and 1.66% on TNSK compared to state-of-the-art models (26). The success is attributed to skip connections, which preserve fine-grained anatomical details, while attention mechanisms enhance boundary features and suppress noise.

Meanwhile, Transformer-based models show emerging potential in capturing long-range dependencies in multimodal thyroid imaging. A fusion model combining ViT with traditional radiomics features, based on dual-modality ultrasound (B-mode and superb microvascular imaging), achieved an AUC of 0.901 for predicting central lymph node metastasis in papillary thyroid carcinoma (107). This outperformed single-modality approaches, demonstrating the value of integrating ViT features with clinical information.

Despite these advances, Transformer-based models face challenges:their high computational demand currently limits widespread clinical adoption, and their performance on limited medical datasets requires careful validation.

### 7.2 Critical barriers to clinical translation

This review highlights that AI, particularly deep learning, exhibits transformative potential across multiple domains of thyroid imaging, including ultrasound, CT, and multimodal fusion analysis. Accumulating evidence demonstrates that AI systems can match or even surpass the diagnostic performance of experienced clinicians while providing clear benefits in optimizing clinical workflows and reducing unnecessary biopsies (108). Nonetheless, a critical paradox remains: despite generally high reported performance metrics, substantial heterogeneity among studies hampers direct comparisons between different models (109).

A thorough appraisal of the literature reveals that observed performance variations arise primarily from methodological disparities rather than inherent algorithmic superiority (109, 110). Several key factors contribute significantly to these discrepancies. First, dataset heterogeneity plays a major role, as variations in composition greatly limit cross-study comparability (109). For instance, models trained on datasets with high malignant nodule prevalence naturally demonstrate different performance characteristics compared to those trained on general screening populations. Furthermore, the underrepresentation of rare subtypes systematically compromises model performance in clinically crucial cases, even when overall accuracy remains high. Second, the rigor of reference standards and validation strategies substantially influences reported outcomes. Performance metrics derived from internal random splits typically exceed those from more robust patient-wise partitions or external multicenter validations, which offer more realistic assessments of generalizability. Studies utilizing postoperative histopathology as the reference standard produce more reliable evaluations compared to those relying solely on cytology (111), particularly for distinguishing challenging follicular lesions. Third, different network architectures possess distinct inductive biases that interact with specific task objectives and dataset characteristics, further complicating cross-study comparisons.

Most current research remains at the technical validation stage, where impressive accuracy metrics often mask fundamental barriers to clinical adoption. A primary challenge involves limited interpretability, as most deep learning models function as “black boxes” that obscure the reasoning behind decisions (112). This opacity can undermine clinical trust when AI recommendations contradict expert judgment, despite emerging techniques like Integrated Gradients and Class Activation Mapping offering partial explanatory capabilities (112).

Publication bias and generalizability concerns present additional hurdles. The literature predominantly features positive results, with unsuccessful or moderate-performance studies remaining underreported, creating overly optimistic perceptions of AI capabilities. Moreover, few models undergo rigorous validation using independent external datasets, casting doubt on their real-world clinical applicability. Clinical workflow integration issues further complicate adoption, as many AI tools are developed without sufficient consideration for seamless incorporation into existing clinical environments. Crucial aspects including user interface design, inference speed, and practical impact on diagnostic efficiency remain inadequately addressed.

Insufficient generalizability constitutes a central barrier to clinical translation. Most models demonstrate strong performance during internal validation but experience significant “performance decay” when applied to out-of-distribution data. This vulnerability stems from multiple sources including patient population diversity, where models developed using homogeneous cohorts frequently show substantially reduced performance when applied to populations with differing demographics, comorbidities, or regional backgrounds. Variations in imaging equipment and acquisition protocols introduce additional domain shifts that challenge model robustness, as differences between manufacturers and operator techniques significantly impact performance. Furthermore, models trained on high-quality tertiary hospital datasets often exhibit markedly diminished diagnostic reliability in primary care or resource-limited settings due to disparities in equipment capability, image quality, and disease spectrum characteristics.

### 7.3 Future directions and translational pathways

To address these challenges and facilitate the translation of thyroid imaging AI from the laboratory to clinical practice, future research should prioritize several key areas. First, data and evaluation standardization requires urgent attention. This involves establishing standardized protocols for data acquisition, annotation, and method reporting. Prospective study designs following frameworks such as IDEAL-AI, combined with multi-institutional collaborations using privacy-preserving technologies like federated learning, will be essential for constructing high-quality datasets that represent real-world clinical diversity.

Second, enhancing model interpretability and reliability must become a priority. Integrating explainable AI into model development and evaluation processes ensures alignment between AI reasoning and clinical decision-making. Beyond conventional accuracy metrics, future studies should incorporate clinically meaningful endpoints, including changes in diagnostic decisions, reductions in unnecessary biopsies, improvements in clinician efficiency, and ultimately, patient outcomes.

Third, external validation and generalizability enhancement deserve greater emphasis. The field needs to shift focus from internal performance optimization to rigorous external multicenter validation. Employing domain adaptation and cross-domain learning techniques will be crucial for improving model robustness across different imaging devices, acquisition protocols, and patient populations.

Finally, clinical trials and workflow integration require systematic investigation. Prospective randomized controlled trials should be conducted to assess the clinical utility of AI tools objectively. Simultaneously, research should explore strategies for seamless integration of these technologies into real-world clinical workflows, ensuring they complement rather than disrupt existing practices.

In conclusion, AI applications in thyroid imaging are at a pivotal stage, transitioning from proof of concept to clinical utility. This review emphasizes that future progress should not merely target incremental gains in accuracy but must also address methodological heterogeneity, model interpretability, and generalizability to establish reliable, trustworthy, and clinically valuable AI systems. Achieving this goal requires sustained interdisciplinary collaboration among clinicians, AI researchers, and industry stakeholders to ensure these technologies are deployed safely, effectively, and equitably, ultimately improving patient care and outcomes in thyroid disease.

## 8 Challenge

While AI technologies demonstrate great potential in thyroid imaging, their development and clinical translation face multiple systemic challenges (113). These obstacles span the entire pipeline from technological development and validation to regulation and clinical integration, necessitating interdisciplinary collaboration and sustained innovation for effective resolution.

### 8.1 Challenges in data acquisition and annotation

The construction of high-quality datasets is fundamental to AI applications, yet significant barriers exist in thyroid imaging. Medical imaging data are highly heterogeneous—variations across device manufacturers (e.g., GE, Siemens, Philips), acquisition protocols, and operators result in low data standardization, directly impacting model training efficacy and generalization capability. Furthermore, high-quality medical data annotation heavily relies on specialists’ expertise and time, making it a resource-intensive process. Substantial inter-observer variability exists during annotation, where disagreements among experts introduce label noise that adversely affects model performance. Strict privacy regulations (e.g., GDPR, HIPAA) further complicate data sharing and aggregation, limiting the scalability of training datasets. For instance, annotating a large-scale dataset for nodule segmentation may require several experienced radiologists devoting hundreds of hours, incurring considerable costs.

### 8.2 Algorithm interpretability and trustworthiness

Although deep learning algorithms exhibit powerful diagnostic and predictive capabilities, their decision-making process is often perceived as a “black box,” significantly hindering their clinical adoption. When a model provides diagnostic recommendations, clinicians may struggle to comprehend its rationale, leading to distrust.

Current interpretability methods can be broadly categorized into two types. Visualization techniques (e.g.,attention mechanisms, class activation mapping) highlight image regions that most influence model decisions, helping clinicians intuitively understand which anatomical features-such as microcalcifications, irregular margins, or hypoechoic areas-drove a particular classification. Feature-based explanation frameworks such as LIME (Local Interpretable Model-agnostic Explanations) and SHAP (Shapley Additive exPlanations) provide quantitative insights into how specific input features contribute to predictions. For example, even if a model accurately predicts the risk of thyroid cancer recurrence, failure to clarify its reasoning —such as identifying which imaging or clinical features contributed to the decision —may impede full clinical acceptance. Despite these advances, significant challenges remain. Current explanation methods have notable limitations: saliency maps can be inconsistent across similar inputs,gradient-based methods may produce noisy or misleading visualizations, and feature-based explanations often struggle to capture complex interactions between features. Moreover, most interpretability techniques have been validated primarily on natural images rather than medical images, and their reliability in clinical contexts remains understudied.

Addressing the “black box” challenge requires a multi-pronged approach extending beyond current methods. One promising direction involves inherently interpretable models that offer an alternative to *post-hoc* explanation. Rather than explaining a black box after the fact,these models are designed to be transparent from the outset. Examples include attention-based networks (e.g., Vision Transformers), where attention weights can be visualized to show which spatial regions the model prioritizes, and rule-based systems that make decisions by comparing inputs to learn exemplars. For thyroid applications, a model that classifies a nodule as malignant because it resembles prototype cases with known malignant features could provide intuitive, case-based explanations that align with clinical reasoning. Another avenue is multimodal explanations that integrate different types of evidence to enhance interpretability. A system might combine visual explanations (heatmaps highlighting suspicious regions), textual justifications (natural-language descriptions of key features), and quantitative comparisons (similarity scores to confirmed cases). This multimodal approach mirrors how radiologists typically communicate findings, describing what they see, where they see it, and how it compares to known patterns. A further critical step involves clinical validation of interpretability methods. Demonstrating that explanations are not only technically valid but also clinically useful requires user studies with practicing clinicians. Key questions include: Do explanations improve diagnostic accuracy when used as decision aids? Do they increase clinician confidence in AI recommendations? Do they help design explainable AI that can enhance human-AI collaboration, but domain-specific validation in thyroid imaging is urgently needed? Looking ahead, future research should prioritize several key directions. Hybrid approaches that combine the performance of DL with the transparency of symbolic AI offer considerable promise. Interactive explainability tools that allow clinicians to query the model and explore counterfactual scenarios (“What if the nodule had smoother margins?”) could foster deeper trust and understanding. Federated explainability techniques that enable explanation generation across institutions without sharing sensitive data address both interpretability and privacy concerns simultaneously. Ultimately, the goal is not merely to open the black box but to create a collaborative framework where AI and clinicians work together, each leveraging their complementary strengths. Achieving this will require close collaboration between AI researchers and clinicians throughout the development and validation process.

Enhancing interpretability requires multifaceted approaches: visualization techniques (e.g., attention mechanisms, class activation mapping) can highlight image regions influential in model decisions, helping clinicians intuitively understand the basis of predictions. Alternatively, interpretability frameworks such as LIME or SHAP can provide feature-based explanations for predictions. For example, even if a model accurately predicts the risk of thyroid cancer recurrence, failure to clarify its reasoning—such as identifying which imaging features contributed to the decision—may impede full clinical acceptance.

### 8.3 Limitations in external validation and generalization

Most current AI model studies remain at the internal validation stage, lacking extensive evaluation across diverse patient populations, imaging devices, and clinical settings. This represents a significant obstacle to clinical translation. Studies have demonstrated that models exhibiting strong performance on internal data may experience a significant decline in performance on external validation sets, a phenomenon often referred to as “performance decay” or “generalization gap” (114, 115). This issue stems from domain shift, as training data often originates from a single institution, specific devices (e.g., high-end ultrasound systems), and relatively homogeneous populations.

This generalization gap stems from three dimensions of data heterogeneity. First, image acquisition variability arises from differences in scanner manufacturers, imaging protocols, and operator expertise. For ultrasound—the most operator-dependent modality—variations in transducer frequency, gain settings, and probe pressure can substantially alter nodule appearance, leading to inconsistent feature extraction across centers. Recent advances in cross-domain segmentation have introduced shape-guided networks that preserve performance across different ultrasound domains by focusing on shape features that are less affected by image acquisition variability. Multicenter studies have further demonstrated that models trained on single-center data may degrade in performance when tested on external datasets, primarily due to differences in image characteristics. Second, population diversity compounds these technical challenges. A model trained on one population, with its specific demographic characteristics (age, ethnicity,body habitus), disease prevalence, and nodule features, may underperform when applied to cohorts with different demographic characteristics. For instance, Chen et al. (30) conducted a multi-center study involving 11,201 ultrasound images from four hospitals, demonstrating that while DL models achieved excellent internal performance (AUC > 0.91),their generalizability across diverse populations required careful validation. Third, annotation inconsistency represents a critical source of heterogeneity. Ground truth labels -whether based on FNAB cytology, histopathology, or expert radiologist consensus—may vary in quality and reliability across centers. Interobserver variability in interpreting ultrasound features has been well documented in thyroid imaging. Even with standardized reporting systems such as TIRADS, which are designed to reduce variability, diagnostic performance remains imperfect. Baek et al. (116) evaluated the DTD-TIRADS system for diffuse thyroid disease and found that while interobserver agreement was nearly perfect (*κ* = 0.81) with the use of standardized criteria, the average diagnostic accuracy was only 82%–84%, with AUCs ranging from 0.86 to 0.88. This suggests that human-level variability, though mitigated by standardization, persists and directly propagates to training labels, potentially confusing models and limiting their upper bound of performance. Models trained on labels from different readers may learn inconsistent patterns, further compounding the generalization challenge.

Multi-center studies have emerged as the gold standard for evaluating and mitigating these effects. Recent large-scale initiatives involving multiple institutions have demonstrated that models trained on diverse, multi-center data achieve significantly better generalizability compared to single-center-trained models (117, 118). However, simply pooling data is insufficient; advanced techniques are required to address remaining challenges (119).

Domain adaptation and generalization methods can reduce the impact of acquisition variability by learning domain-invariant features. For instance, Zhang et al. (120) proposed a shape-guided network that leverages task-relevant but domain-independent features, such as nodule shape, to improve cross-domain segmentation performance across six ultrasound domains without relying on extensive data augmentation.

Multi-task learning approaches that incorporate clinical knowledge have shown promise in improving model robustness across different datasets. Gao et al. (121) developed a clinical knowledge embedded method based on multi-task learning that integrates ACR TI-RADS features, achieving an AUC of 0.917 on external test sets and significantly outperforming single-task baselines.

Multi-modal fusion strategies that combine information from different imaging techniques can provide complementary features that are less susceptible to single-modality variability. Li et al. (122) proposed a dual-modality watershed fusion network that integrates ultrasound with dynamic vascular features from contrast-enhanced ultrasound video, achieving an AUC of 0.920 for thyroid nodule classification.

Comprehensive model comparison and selection frameworks are essential for identifying architectures that generalize well. Zhang et al. (123) systematically reviewed deep learning methods for thyroid nodule classification, comparing architectures including ResNet variants (ResNet-18 achieving 98.4% accuracy), ensemble methods, and Vision Transformers, and highlighted the wide range of reported performance metrics and the need for standardized evaluation protocols.

When deployed in various healthcare settings (e.g., primary care hospitals), with alternative machines (e.g., those from other manufacturers), or within diverse demographic groups (e.g., different regions or age groups), model performance may degrade significantly. Addressing this requires rigorous validation using independent, multicenter, multi-device, and demographically diverse test sets, alongside techniques such as domain adaptation and federated learning to improve robustness across data distributions.

### 8.4 Variability in global healthcare systems

Beyond technical and methodological hurdles, the translation of AI into clinical practice is profoundly influenced by the vast heterogeneity of healthcare systems worldwide. These disparities create divergent sets of challenges that must be addressed for equitable and effective global deployment. The feasibility of AI implementation varies dramatically across settings. High-resource environments in tertiary care centers are characterized by state-of-the-art imaging equipment, integrated systems, and a wealth of specialist expertise. Here, the primary challenges involve ensuring seamless system interoperability, optimizing workflows, and establishing effective reimbursement models. In stark contrast, low and middle-resource settings often contend with older or basic imaging devices, limited digital infrastructure, and a critical shortage of radiologists and endocrinologists. In these contexts, the challenges are fundamentally different: AI models must function reliably on lower-quality images and be deployable on computationally limited hardware (e.g., portable ultrasound devices). Furthermore, the role of AI shifts from being an assistant to a potential “expert extender,” raising the stakes for its reliability and the need for designs that account for different user profiles.

### 8.5 Regulatory and approval hurdles

AI-based medical devices face stringent regulatory approval processes (e.g., the U.S. FDA, CE marking in the EU, and China's National Medical Products Administration), which demand comprehensive clinical validation to demonstrate safety, efficacy, and generalizability. Key regulatory challenges include the need for costly and time-consuming multicenter prospective clinical validations, defining acceptable thresholds for performance decay, and establishing dynamic regulatory frameworks for continuously learning algorithms to address issues arising from iterative model updates.

### 8.6 Clinical integration and practical barriers

Integrating AI tools into existing clinical workflows poses numerous practical difficulties (124). Most AI systems are developed as standalone platforms with limited integration into hospital systems, which can lead to workflow disruptions. For instance, clinicians may need to export images from picture archiving and communication systems, upload them to a separate AI platform, wait for analysis results, and manually enter findings into reporting systems—ultimately reducing efficiency. Moreover, adopting AI tools requires changes to physicians’ conventional practices, encountering user acceptance and training challenges. Clinicians of different ages and digital literacy levels vary in their receptiveness to new technology, necessitating tailored training and support. Medico-legal concerns also remain unresolved: in cases where an AI system provides an erroneous diagnosis leading to adverse outcomes, the allocation of responsibility among physicians, hospitals, algorithm developers, and device manufacturers lacks a clear legal framework.

### 8.7 Ethical and social considerations

The application of AI in healthcare raises several ethical issues. Algorithmic bias is particularly concerning: if training data lack diversity (e.g., underrepresentation of certain ethnicities, age groups, or disease subtypes), models may perform poorly for these populations, exacerbating healthcare disparities. Data privacy and security are also critical, as medical data contains highly sensitive information that must be rigorously protected throughout collection, storage, and processing (125). Furthermore, introducing AI may alter physician–patient relationships, where overreliance on technology could undermine clinical expertise and patient trust. Maintaining a “human-in-the-loop” approach—balancing technological assistance with professional judgment—requires careful consideration and deliberation.

Addressing these challenges requires a multipronged approach: developing large-scale, multicenter datasets using privacy-preserving technologies like federated learning; designing more robust and interpretable algorithm architectures; advancing regulatory science tailored to AI-based medical devices; and creating integrated solutions centered on clinical workflows with seamless interoperability into existing hospital systems. Only through interdisciplinary collaboration can we systematically overcome these technical, regulatory, and ethical barriers, unlocking AI's potential to enhance diagnostic accuracy, optimize workflows, and improve patient outcomes in thyroid imaging.

## 9 Conclusion

In conclusion, the application of AI in the diagnosis and management of thyroid diseases presents significant advancements and opportunities for enhancing clinical outcomes. AI, particularly through ML and DL algorithms, has demonstrated notable success in improving diagnostic accuracy in imaging modalities such as ultrasound, CT, and SPECT. These technologies facilitate the automatic detection, classification, and segmentation of thyroid nodules, providing clinicians with tools that enhance diagnostic consistency and efficiency.Integrating AI can potentially reduce human error, improve early detection of malignant nodules, and optimize personalized treatment strategies.

Despite these promising developments, challenges remain in data acquisition, model validation, and the interpretability of AI systems, which are essential for ensuring trustworthiness in clinical practice. Looking forward, several critical directions warrant prioritized attention from the research community. Large-scale, multicenter, prospective trials are urgently needed to validate AI models across diverse populations, imaging devices, and clinical settings, moving beyond the retrospective, single-center studies that dominate the current literature. Such trials adhere to reporting guidelines, such as CONSORT-AI, to ensure reproducibility and clinical credibility. The evolving landscape of AI-based medical devices, particularly continuously learning algorithms, calls for dedicated investigation into regulatory science and approval pathways. Collaborative efforts between researchers, regulators, and industry stakeholders are essential to develop adaptive frameworks that balance innovation with patient safety.

Addressing algorithmic fairness and bias is equally imperative. Models trained predominantly on specific populations may underperform in underrepresented groups, potentially exacerbating healthcare disparities. Future research must systematically evaluate model performance across demographic subgroups and develop techniques to detect and correct bias. The pursuit of explainable AI extends beyond technical development to include rigorous clinical validation. User studies with practicing clinicians are needed to verify that interpretability methods genuinely enhance trust and decision-making in real-world clinical contexts.

Implementation science deserves greater attention, as even clinically effective AI tools may fail if they disrupt workflows, require excessive training, or lack clear value propositions. Research should examine the real-world impact of AI integration on clinical workflows, clinician satisfaction, and cost-effectiveness, factors that ultimately determine whether promising technologies achieve widespread adoption.

International collaboration and data sharing frameworks, such as federated learning initiatives that enable model training across institutions without sharing raw data, could dramatically accelerate progress toward generalizable, equitable AI systems. Combined with open-source benchmark datasets that capture the full spectrum of acquisition and population variability, these efforts will help bridge the gap between algorithmic innovation and clinical impact.

With continued technological advancements, robust data collection efforts, and collaborative research to overcome these limitations, AI holds great potential to transform thyroid disease diagnostics by offering a more precise, efficient, and personalized approach to patient care, ultimately improving patient outcomes and enabling more efficient healthcare delivery systems.
